# 贝伐珠单抗治疗非小细胞肺癌的相关不良反应及处理原则

**DOI:** 10.3779/j.issn.1009-3419.2010.06.001

**Published:** 2010-06-20

**Authors:** 刚 程, 力 张

**Affiliations:** 1 100730 北京，卫生部北京医院肿瘤内科 Department of Medical Oncology, Beijing Hospital, Beijing 100730, China; 2 510060 广州，中山大学肿瘤防治中心 Cancer Center, Sun Yat-Sen University, Guangzhou 510060, China

一个世纪以前，研究者发现肿瘤的生长伴随着血管化程度而增加。随着研究的深入，人们认识到血管生成是肿瘤发展的关键步骤，并提出了抑制血管生成以治疗肿瘤的新策略^[1, 2]^。血管内皮生长因子(vascular endothelial growth factor, VEGF)是最重要的促血管生成因子，广泛表达于人体组织，主要通过与内皮细胞表面的VEGF受体2(VEGFR2)结合，激活下游通路的级联效应而发挥其促血管生成作用，效应包括：促进内皮细胞增殖；动员骨髓中内皮祖细胞进入循环并归巢、分化、分裂形成新血管；增加血管通透性等。VEGF在成人中的正常生理作用有限并受到高度调控，但肿瘤细胞可大量分泌VEGF并促进肿瘤血管的生成。血管靶向药物贝伐珠单抗(Bevacizumab)是一种人源化单抗，能特异性地结合VEGF分子并阻断其作用。基于一系列临床研究验证的生存获益，美国食品药品监督管理局(FDA)于2004年首次批准贝伐珠单抗用于治疗转移性结直肠癌；2007年批准其用于诊疗晚期非小细胞肺癌^[3]^。作为第一个在全球获准上市的抗血管生成的单抗药物，目前在全球市场贝伐珠单抗已先后获准用于诊疗晚期结直肠癌、肺癌、乳腺癌、肾癌、恶性胶质瘤等，已用患者超过50万例。在晚期非小细胞肺癌治疗领域，其与化疗联合已成为一线标准方案之一。国外众多的临床研究已显示，贝伐珠单抗的严重不良反应发生率低，毒性与化疗药物无重叠，但作为一种全新的肿瘤治疗策略，国内临床医生对其特殊不良事件的经验尚少。借2010年贝伐珠单抗在中国上市之际，本文就其治疗晚期非小细胞肺癌的相关不良反应、发生机制及可能的预防和处理原则进行简单综述。

## 1 高血压

高血压是贝伐珠单抗治疗各种实体瘤中常见的相关不良反应，发生率为8%-67%，其中3级以上严重高血压的发生率为5%-18%^[3]^，临床研究中尚未发现因高血压致死的病例报道。在非小细胞肺癌的Ⅲ期研究E4599和AVAiL^[4, 5]^中，贝伐珠单抗联合化疗组CTCAE 3级以上高血压的发生率为6%-9%，在更接近真实临床应用环境的Ⅳ期研究SAiL^[6]^中，3级以上高血压发生率仅为0.4%。在贝伐珠单抗联合化疗治疗非小细胞肺癌的中国研究中，3级以上高血压发生率为3%^[7]^。在这些关键性Ⅲ期或Ⅳ期临床试验中，未发现年龄、剂量与高血压发生率的相关性；但基于已发表文献数据的荟萃分析^[8]^表明，在结直肠癌、肺癌、乳腺癌、肾癌等多种实体瘤中，高血压可能与贝伐珠单抗呈剂量依赖性：与对照组相比，单次剂量为3 mg/kg、5 mg/kg、7.5 mg/kg的低剂量组发生高血压的相对风险比(relative ratio, RR)为3.0(95%CI: 2.2-4.2, *P* < 0.001)；而单次剂量为10 mg/kg、15 mg/kg的高剂量组的RR为7.5(95%CI: 4.2-13.4, *P* < 0.001)。

贝伐珠单抗引起高血压的机制尚未完全明确。有研究^[9-11]^认为正常情况下VEGF通过促进内皮细胞产生血管舒张剂一氧化氮(nitric oxide, NO)和前列环素(prostacydin, PGI_2_)从而在维持血压稳态方面发挥作用，抑制VEGF后NO和PGI_2_的产生减少可引起高血压。也有研究认为其引起高血压的机制可能与微循环的改变有关：Mourad等^[12]^研究了20例结直肠癌患者，发现贝伐珠单抗治疗6个月后，皮肤毛细血管密度显著降低，内皮细胞功能性反应显著下降，这些改变可导致外周循环的阻力增加，从而引起血压升高。总体来说，贝伐珠单抗高血压的发生率较同为抗血管生成类药物的VEGF受体酪氨酸激酶抑制剂(VEGF receptor tyrosine kinase inhibitor, VEGFR TKI)相对低，这可能与VEGFR的血管活性功能有关。从应用贝伐珠单抗的临床经验中可以发现，即使在同一类型肿瘤中，不同临床研究所报道的高血压发生率也有较大差异；一些病人在应用贝伐珠单抗数分钟后就可发生高血压，一些病人在数天或数周之后发生，而另一些病人则不会发生高血压，且发生的时间似乎与伴随的化疗方案无关，这说明贝伐珠单抗引起高血压的机制存在较大的个体差异。此外，有报道显示高血压的发生可能与贝伐珠单抗的疗效相关：SAiL研究的回顾性分析^[13]^显示，治疗后发生高血压的患者的总生存期较未发生高血压的患者长(18.8个月 *vs* 12.9个月)。

贝伐珠单抗相关高血压的处理原则包括：①贝伐珠单抗不宜应用于未控制的高血压患者，用药前需监测基线血压。对于既往有高血压病史者，开始治疗前血压应控制在150 mmHg/100 mmHg以下，对于已有高血压并发症的患者(如脑血管意外、肾病等)可能需更严格的控制；②用药期间密切监测血压。虽然贝伐珠单抗引起的高血压多发生于治疗开始的1年内，但对于治疗期间曾发生高血压或原有高血压加剧的患者，在治疗停止之后仍应规律性地监测血压；③目前尚无证据表明对贝伐珠单抗引起的高血压，某一类的降压药物疗效更好，因此对高血压患者采取常规降压药物进行治疗即可；④若患者发生中度以上的高血压(收缩压高于160 mmHg，舒张压高于100 mmHg)，应暂停贝伐珠单抗并给予降压治疗，直至血压恢复到治疗前水平或低于150 mmHg/100 mmHg方可恢复贝伐珠单抗治疗；⑤若患者的高血压经治疗1个月仍未控制或发生高血压危象，应永久停用贝伐珠单抗。

## 2 蛋白尿

蛋白尿是贝伐珠单抗联合化疗治疗多种肿瘤时另一常见的相关不良反应，发生率约为0.7%-38%^[14]^，但大部分为无症状性蛋白尿，3级蛋白尿的发生率 < 3%，4级蛋白尿(肾病综合征)的发生率 < 1%^[3]^。在非小细胞肺癌的E4599、AVAiL和SAiL研究^[4-6]^中，贝伐珠单抗联合化疗3级以上蛋白尿的发生率为0.1%-3%，在贝伐珠单抗联合化疗治疗晚期非小细胞肺癌的中国研究^[7]^中，3级以上蛋白尿发生率为8.6%。荟萃分析^[8]^显示蛋白尿可能与贝伐珠单抗呈剂量依赖性：与对照组相比，低剂量组的RR为1.4(95%CI: 1.1-1.7, *P*=0.003)；高剂量组RR为2.2(95%CI: 1.6-2.9, *P* < 0.001)。

贝伐珠单抗导致蛋白尿的原因部分与其引起的高血压相关^[15]^，但这两种副反应并不总是相伴或相继发生的，提示蛋白尿的产生还有其它原因。Eremina等^[16]^对6例患者的肾活检揭示贝伐珠单抗引起的蛋白尿患者中存在肾小球血栓性微血管病，并通过动物实验进一步推断，肾小球足细胞所分泌的VEGF是维持肾小球内皮细胞正常结构和功能所必需的，具体可能是通过上调抗凋亡基因如*Bcl-2*的表达、增加NO的生成、诱导衰变加速因子(decay accelerating factor, DAF)的表达等途径，对内皮细胞起到保护作用。当贝伐珠单抗抑制了VEGF对内皮细胞的保护作用，可致肾小球的滤过通透性增高、重吸收能力降低，最终形成蛋白尿。

贝伐珠单抗相关蛋白尿的处理原则包括：①每次给予贝伐珠单抗之前进行尿蛋白试纸检查。尿蛋白试纸1+：继续按疗程给予贝伐珠单抗，无需特别处理；②若尿蛋白试纸2+：给予贝伐珠单抗，并在下次贝伐珠单抗给药前3天检测24 h蛋白尿；③下次给予贝伐珠单抗前，24 h尿蛋白≤2 g：给予贝伐珠单抗，并在每次给予贝伐珠单抗前检测24 h尿蛋白，直至尿蛋白低于1 g，则可改为尿试纸随访。24 h尿蛋白 > 2 g：暂停贝伐珠单抗，并在下次贝伐珠单抗给药前检测24 h尿蛋白，直至24 h尿蛋白 < 2 g后方可继续给予贝伐珠单抗。如果24 h尿蛋白≥2 g持续时间 > 3个月，则永久终止贝伐珠单抗治疗；④尿蛋白4+或3+：暂停贝伐珠单抗并检测24 h尿蛋白。如果24 h尿蛋白 < 2 g，按计划给予贝伐珠单抗；如果24 h尿蛋白≥2 g的持续时间 > 3个月，则永久终止贝伐珠单抗；⑤肾病综合症：永久终止贝伐珠单抗；⑥终止贝伐珠单抗治疗后仍应每3个月检测一次24 h尿蛋白，直到24 h尿蛋白 < 1 g。

## 3 血栓栓塞

贝伐珠单抗联合化疗治疗的肿瘤患者发生血栓栓塞性疾病的风险增加。最常见的动脉血栓事件(arterial thromboembolic events, ATE)是心脑血管事件，在已批准的多种适应症的临床研究^[3]^中，贝伐珠单抗联合化疗组3级以上ATE的发生率为2.6%，而单用化疗组(对照组)为0.8%，ATE的危险因素是曾发生过ATE(*P* < 0.001)或年龄65岁以上(*P*=0.01)^[17]^。静脉血栓事件(venous thromboembolic events, VTE)包括深静脉血栓和肺栓塞。在贝伐珠单抗联合化疗治疗晚期非小细胞肺癌的AVAiL研究^[5]^中，对照组和试验组的3级以上VTE发生率分别为6%和7%，在Ⅳ期SAiL研究^[6]^中，3级以上血栓栓塞事件的总发生率为4.8%。一项对贝伐珠单抗联合化疗治疗各类实体瘤的临床研究中的7 956例患者进行的荟萃分析^[18]^显示，贝伐珠单抗联合化疗组所有级别和3级以上VTE的发生率较对照组分别提高了11.9%和6.3%，低剂量组和高剂量组的风险相似。在贝伐珠单抗联合化疗治疗晚期非小细胞肺癌的中国研究^[7]^中，3级以上血栓栓塞事件的发生率仅为0.5%，显示亚洲人群在此情况下静脉血栓事件的风险似乎低于白种人。

贝伐珠单抗引起血栓栓塞事件的原因有：VEGF是内皮细胞最重要的增殖和保护分子，该信号通路的抑制降低了内皮的防御和修复能力，使内皮表面完整性丧失、基膜下胶原暴露、组织因子激活；此外，VEGF通路促进血小板抑制剂NO和PGI_2_的产生，贝伐珠单抗抑制VEGF后促进了血小板的聚集。值得指出的是，恶性肿瘤患者本身具有血栓栓塞倾向，贝伐珠单抗的应用仅轻微地增加了其风险，临床应用时应针对其风险和获益进行综合衡量决定。

贝伐珠单抗相关血栓栓塞事件的处理原则包括：①有ATE病史的患者慎用贝伐珠单抗，年龄 > 65岁的老年患者应用时注意血栓栓塞事件的监测。②一旦发生任何级别的ATE，终止应用贝伐珠单抗。③如发生3级以上深静脉血栓，暂停贝伐珠单抗2周，若2周后抗凝疗效稳定、未发生3级以上出血事件且肿瘤未侵犯主要血管，可重新开始贝伐珠单抗治疗。④若发生肺栓塞，采用相应对症治疗并停用贝伐珠单抗。

## 4 出血

贝伐珠单抗联合化疗治疗非小细胞肺癌的出血分为两类，一类是非肿瘤相关的轻度皮肤粘膜出血，最常见的是1级鼻出血。在Ⅲ期E4599研究^[4]^中，贝伐珠单抗联合化疗组3级以上鼻出血率为0.7%，3级以上胃肠道出血率为0.9%。另一类是肿瘤相关的出血，即肺出血和咯血。在一项早期的肺癌Ⅱ期研究^[19]^中，贝伐珠单抗联合化疗组有9%的患者发生严重咯血，使咯血成为贝伐珠单抗治疗肺癌时令人关注的副反应。经分析，组织学鳞癌可能是发生大咯血的危险因素，因此，之后贝伐珠单抗联合化疗治疗非小细胞肺癌的研究均将鳞癌作为排除标准，并进一步排除了肿瘤侵犯大血管的患者，使咯血发生率明显下降。在全球2 166例晚期非小细胞肺癌患者中进行的Ⅳ期SAiL研究^[20]^显示，贝伐珠单抗联合化疗所有级别的肺出血和咯血发生率为7.9%，且其中3级以上的发生率仅为0.6%。在中国进行的贝伐珠单抗联合卡铂和紫杉醇(TC)方案治疗非小细胞肺癌的研究^[7]^中，3级以上咯血发生率为0.5%。

值得注意的是，在所有的贝伐珠单抗治疗非小细胞肺癌的临床研究中，均排除了基线状态有脑转移的患者，但在治疗期间部分病人仍会出现脑转移，这部分病人的脑出血率成为令人关心的问题。一项分析综合了贝伐珠单抗联合化疗治疗多种实体瘤的13项随机对照研究、2项大样本Ⅳ期研究和2项允许经治疗后脑转移患者入组的研究^[21]^发现，贝伐珠单抗治疗的脑转移灶出血率低(0.8%-3.3%)，与历史对照数据接近。针对在一项存在经治脑转移灶的晚期肺癌患者的研究^[22]^中，贝伐珠单抗与化疗或厄洛替尼联用的115例患者均未发生2级以上脑转移灶出血。目前，欧盟已解除了脑转移灶作为贝伐珠单抗联合化疗应用的禁忌症，美国FDA则从无此禁忌症。

此外，由于肿瘤病人常合并有抗凝治疗，这部分病人在初期也被排除在贝伐珠单抗联合化疗的临床研究之内。但最近的一项大样本Ⅳ期研究发现，贝伐珠单抗同时应用抗凝治疗并不增加3级以上出血事件的风险。

贝伐珠单抗致出血的原因与血栓栓塞事件有共同之处，即与抑制VEGF导致的内皮细胞正常功能紊乱有关。此外，由于肿瘤血管增生紊乱、结构异常，在贝伐珠单抗治疗后周围肿瘤组织退缩明显、形成空洞时，可能造成血管结构失去支撑、易于出血。因此，选择适合的患者对于防止严重肺出血/咯血具有重要的意义。处理原则包括：①排除具有组织学鳞癌、肿瘤紧邻或侵犯大血管、基线肿瘤空洞形成等危险因素的患者。②排除近期(3个月内)有咯血史(≥1/2茶匙红色血液，约2.5 mL)的患者。③出血体质或后天凝血功能障碍的患者慎用贝伐珠单抗。④治疗期间密切监测凝血指标和出血情况。⑤治疗后发生肺出血/咯血的患者可考虑停用贝伐珠单抗。

## 5 胃肠道穿孔

贝伐珠单抗联合化疗治疗各类实体瘤的胃肠道穿孔发生率为0.3%-2.4%^[3]^。一项综合了17项随机对照研究中12 294例患者的荟萃分析^[23]^表明，接受贝伐珠单抗治疗患者的胃肠道穿孔风险增高，穿孔的总发生率为0.9%(95%CI: 0.7-1.2)，相对于对照组的RR为2.14(95%CI: 1.19-3.85)，穿孔患者的死亡率为21.7%(95%CI : 11.5-37.0)。大部分病例在贝伐珠单抗治疗的50天内发生，严重程度表现不一，轻者无任何症状，仅通过影像学检查发现；严重者可伴有腹部脓肿、瘘管形成。穿孔的发生率与肿瘤类型和剂量相关，结直肠癌和肾癌患者的风险最高，胰腺癌最低。在中国进行的一项有关非小细胞肺癌的临床研究^[7]^中，贝伐珠单抗联合化疗的胃肠道穿孔事件的发生率为0.5%。

胃肠道穿孔的发生机制被认为可能与其对微循环的抑制作用导致胃肠道局部缺血有关；尤其是在结直肠癌患者或本身存在胃肠道器质性病变的基础上，贝伐珠单抗可能会抑制病变的愈合，并加剧其发展。

虽然胃肠道穿孔的发生率较低，但由于其死亡率较高，因此在临床应用中应引起重视。①腹腔炎症、肿瘤坏死、憩室炎、化/放疗相关结肠炎、既往胃肠道梗阻/穿孔史、高龄患者(可能伴有合并疾病如憩室或腹部血管的动脉狭窄)的患者慎用贝伐珠单抗。②在整个治疗期间加强监测，如果患者出现腹痛等症状，在进行鉴别诊断时应考虑胃肠道穿孔的可能。③一旦出现胃肠道穿孔，立即给予相应治疗并永久停用贝伐珠单抗。

## 6 伤口愈合综合症

伤口愈合综合症主要指伤口裂开或延迟愈合。贝伐珠单抗对伤口愈合的影响主要在结直肠癌的研究中进行了分析：在贝伐珠单抗联合化疗治疗期间接受手术患者的伤口愈合综合症发生率明显高于对照组(15% *vs* 4%)^[3]^。

显而易见，伤口愈合综合症的发生机制与贝伐珠单抗对伤口愈合过程中的新生血管的抑制作用相关。因此，目前推荐对发生需要治疗的伤口愈合综合症患者暂停贝伐珠单抗；对择期手术的患者，手术前后各1个月内不推荐使用贝伐珠单抗；对急诊手术的患者则应进行风险受益的评估后决定。

## 7 充血性心力衰竭(congestive heart failure, CHF)

在贝伐珠单抗联合化疗治疗非小细胞肺癌的SAiL研究^[6]^中，有4.8%的患者发生CHF，其中3级以上的发生率为1.0%。在中国进行的贝伐珠单抗联合化疗治疗晚期非小细胞肺癌临床研究中，未见CHF的发生。CHF风险增高的机制可能与抑制VEGF引起的心肌微血管网减少趋势有关，与其它心脏毒性药物的联合使用也可能使风险增高。因此，有6个月内发生过心血管意外/心肌梗死病史，有不稳定型心绞痛、严重心功能不全(NYHA≥2级)、需要药物治疗的严重心律不齐的患者不宜使用贝伐珠单抗。在临床应用中应注意仔细询问病史，并密切监测心功能。

## 8 胃肠外瘘管形成

严重的胃肠外瘘管形成包括气管-食管瘘、支气管-胸膜瘘、胆道、阴道、膀胱等部位瘘，贝伐珠单抗治疗的患者发生胃肠外瘘管形成的风险较对照组有所升高，但总体较为少见，在各项临床研究中的发生率低于0.3%^[3]^，大部分发生在治疗的前6个月内。Spigel等^[24]^报道的共包括34例小细胞肺癌和非小细胞肺癌患者的两项Ⅱ期研究中，发生4例确认的和1例可疑的气管-食管瘘，导致入组的终止和FDA说明书的更改。这些患者的共同点是同时接受了化疗和放疗，且大多有食管炎病史。有推测这些气管-食管瘘的发生机制可能与抑制VEGF导致的食管基础性疾病愈合困难或加剧，以及化疗药物的放疗增敏作用相关。因此，在贝伐珠单抗治疗前，应注意询问病史，以及食管镜、胃镜、阴道镜、膀胱镜等检查史，治疗期间注意监测。发生内脏器官瘘管的患者应停用贝伐珠单抗。

## 9 可逆性后部白质脑病综合症(reversible posterior leukoencephalopathy syndrome, RPLS)

这是贝伐珠单抗一种少见的副反应，在各项临床研究中的发生率 < 0.1%^[3]^，可在开始治疗后16 h至1年内发生，表现为头疼、抽搐、意识不清、视物模糊甚至视力丧失等视觉和神经精神症状。发生原因不明。发生RPLS的患者应停止使用贝伐珠单抗，之后一般可自行缓解，但也有一小部分患者的症状可持续。

## 10 总结

综上所述，贝伐珠单抗的不良反应经过了大量的临床研究和实际应用，已经得到了较全面的阐明。由于其独特的作用机制，贝伐珠单抗的不良反应与一般化疗药物具有不同之处，临床医生在使用该药物时应该注重观察并及时处理这些不良反应。一些较常见的贝伐珠单抗相关不良反应如高血压、蛋白尿等均可通过常规的方式得到控制；一些较不常见但后果较严重的不良反应如咯血、胃肠道穿孔等可通过选择合适的病人加以避免。另一方面，这些毒副作用与常规化疗的不良反应又不相互重叠，对化疗药物本身的副反应影响小，因此，与化疗药物联合应用的安全性比较高。我们有望通过不良反应的有效预防和管理，更好地发挥贝伐珠单抗为合适的肺癌患者带来的生存获益。
